# A data set of global river networks and corresponding water resources zones divisions v2

**DOI:** 10.1038/s41597-022-01888-0

**Published:** 2022-12-15

**Authors:** Denghua Yan, Chenhao Li, Xin Zhang, Jianwei Wang, Jianming Feng, Biqiong Dong, Jingjing Fan, Kun Wang, Cheng Zhang, Hao Wang, Jianyun Zhang, Tianling Qin

**Affiliations:** 1 State Key Laboratory of Simulation and Regulation of Water Cycle in River Basin, China Institute of Water Resources and Hydropower Research, No. 1 Fuxing Road, Haidian District, Beijing, 100038 China; 2 State Key Laboratory of Hydrology-Water Resources and Hydraulic Engineering, Nanjing Hydraulic Research Institute, Nanjing, 210029 China

**Keywords:** Hydrology, Hydrology

## Abstract

The scale and topological relationship of river networks (RN) and water resources zones (WRZ) directly affect the simulation results of global multi-scale hydrological cycle and the accuracy of water resource refined evaluation. However, few existing global hydrological data sets take account of both aspects simultaneously. Here, we constructed a new hydrologic data set with a spatial resolution of 90 m as an upgraded version of the GRNWRZ V1.0. This data set had proper grading and partitioning thresholds and clear coding of topological relationships. Based on maintaining the accuracy of river networks in the GRNWRZ V1.0, we determined the more refined thresholds and created a new coding rule, which made the grading RN and partitioning WRZ more precise and the topological relationship more intuitive. Supported by this data set, the accuracy and efficiency of the large-scale hydrological simulation can be guaranteed. This data set provides fundamental data support for global water resources governance and global hydrological modeling under climate change.

## Abstract (summary)

**Table:** 

Measurement(s)	river • stream • drainage basin • watershed
Technology Type(s)	computational modeling technique
Sample Characteristic - Environment	freshwater river
Sample Characteristic - Location	Earth (Planet)

## Background & Summary

Under the influence of global climate change and anthropogenic activities, significant changes have undergone in terrestrial water storage and their spatial distribution^1,2^. To clarify these changes, global multi-scale hydrological simulation and refined water resources evaluation are vital^3,4^. High-precision river network (RN) and water resources zone (WRZ) are the two key basic data for the abovementioned simulation and evaluation. In particular, the accuracy and refinement of the RN and the WRZ directly determine the relationship of regional runoff generation and flow concentration, affecting the efficiency of large-scale hydrological simulations^5,6^. Meanwhile, grading RN and partitioning WRZ are closely related to the evaluation of runoff-typed water resources and generalized water resources^7^, affect the identification of regional water resources endowments, and indirectly determine the allocation plan of water resources and the water constraint of ecological restoration. Therefore, constructing a complete and detailed global RN and WRZ data set is a hot issue at the forefront of climate change, hydrology, water resources, and ecological environment.

As the spatial resolution of the digital elevation model (DEM) improves, many hydrological data sets with high spatial resolution have appeared. In 2000, the Earth Resources Observation and Science (EROS) Center of the United States Geological Survey (USGS) developed a data set named HYDRO 1K^8^, containing stream lines and basin boundaries based on the global 30 arc-seconds DEM (GTOPO30). The World Wildlife Fund (WWF) in association with the USGS, the International Centre for Tropical Agriculture (CIAT), the Nature Conservancy (TNC), the McGill University, the Australian National University, and the Center for Environmental Systems Research (CESR) have jointly developed the hydrological data and maps based on SHuttle Elevation Derivatives at multiple Scales (HydroSHEDS)^9^ during 2006 to 2009. The spatial resolution ranges from 3 arc-seconds to 30 arc-seconds. It is superior to other similar data sets at that time and provides essential data support for enhancing river flow modeling^10^. The HydroSHEDS v2, which improves upon the quality and limitations of HydroSHEDS v1, will be released in 2023. In 2017, the USGS developed the Hydrologic Derivatives for Modeling and Applications (HDMA)^11^ database based on HydroSHEDS, Global Multi-resolution Terrain Elevation Data 2010 (GMTED2010), and the Shuttle Radar Topography Mission (SRTM)^12^ in cooperation with NASA Goddard Space Flight Center. Its spatial resolution ranges from 3 arc-seconds (for most of the globe south of 60° N) to 7.5 arc-seconds (for the areas north of 60°N). The spatial resolution of the hydrological data set improved again. In 2019, Yamazaki *et al*. developed a global hydrological dataset at 3 arc-seconds resolution (~90 m at the equator) named MERIT Hydro^13^ based on MERIT DEM^14^ and multiple inland water maps. It contains flow direction, flow accumulation, hydrologically adjusted elevations, and river channel width, which have been used in some recent river network studies^15,16^. HydroALTS^17^, which is derived from the HydroSHEDS core product, correlates hierarchically nested sub-basins, as well as individual river reaches with hydro-environmental characteristics, providing 56 variables in 6 categories.

Although the spatial resolution of existing hydrological data sets has been improved dramatically, which helps to enhance the results accuracy and scales refinement in water resources evaluation, there are some deficiencies. Firstly, it is difficult to automatically generate correct digital rivers using original DEM data, thus affecting the regional flow concentration relationship. This is due to the low vertical resolution^18^ or some observation gaps in high-relief mountains and over water bodies of the original data^14^, which makes it impossible to judge the correct flow direction of natural rivers on the land surface without or with very little auxiliary data. Secondly, the basins of many data sets are divided and coded using the Pfafstetter coding system^19^, which limits the number of sub-basins to no more than 9^20,21^. It fails to highlight the details of the flow concentration relationship among sub-basins. Finally, the coding method of reaches and their zones is complex and does not directly correspond to the river spatial network structure. It also requires a correlation or calculation between fields to obtain the upstream and downstream relationships among basins.

In response to the above problems, we developed a data set entitled ‘A data set of global river networks and corresponding water resources zones’ (GRNWRZ V1.0)^22^ with a spatial resolution of 90 m based on the SRTM and the ASTER Global Digital Elevation Model (ASTER GDEM)^23^ in 2019. The RN in this dataset has been extensively verified manually in combination with natural rivers in Google Earth, and it is relatively accurate compared to other data sets, especially in plain and inland areas^24^. Each basin is divided into 99 sub-basins maximumly through the coding method determined according to the stem-branch topology, which solves the problem of the upper limit of the number of sub-basins. At the same time, the code numbers of reaches and corresponding sub-basins are unified to ensure that both have the same code number. However, GRNWRZ V1.0 still has some limitations for application in the more widely used sub-basin based distributed hydrological models (e.g., SWAT^25,26^, WEP-L^27^). Firstly, hydrological simulation and water resources evaluation of super-large WRZ (>10000 km^2^) and ultra-small WRZ (<100 km^2^) are hindered. For super-large WRZ, many small sub-basins in the main stream reaches do not match their level in GRNWRZ V1.0, affecting the efficiency of the hydrological simulation. For ultra-small WRZ, the flow concentration relationship between sub-basins is unclear, affecting the accuracy of water resources evaluation results. Secondly, the flow concentration relationship represented by the code numbers among sub-basins in the coastal region is not emphasized. Lastly, GRNWRZ V1.0 does not consider that those exorheic rivers eventually flow to different oceans when they define rivers, affecting global terrestrial water resources estimation.

The paper proposes a partitioning and coding method that addresses known limitations in GRNWRZ V1.0^24^ while retaining its advantages. Based on this method, we used the SRTM, the ASTER GDEM, and the high-precision river network data in GRNWRZ V1.0 to construct a data set of global RN and WRZ at levels 1 to 7 (excluding Greenland and Antarctica) at three arc-seconds resolution based on these methods mentioned above, entitled ‘A data set of global river networks and corresponding water resources zones divisions V2.0.’ (GRNWRZ V2.0). It can meet the needs of high-precision and multi-scale hydrological simulation and water resources evaluation.

## Methods

**Fig. 1:**
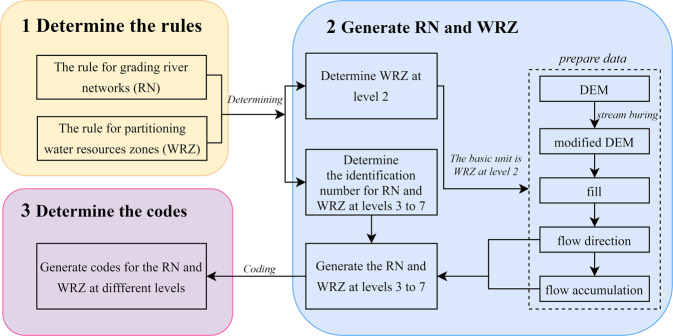
The process of generating and coding the global digital RN and WRZ.

We define the levels of RN and WRZ from levels 1 to 7, which are based on comprehensive consideration of the basin area and whether the river system connections within WRZ can be fully highlighted. On this basis, we establish a new coding rule to clarify the topological relationship. Meanwhile, to ensure the consistency of codes for RN and WRZ, we have stipulated the codes’ length for each level. It can ensure the continuity and effectiveness of codes. The construction method and data generation process of RN and WRZ are summarized in Fig. 1.

### Determine the rules for grading RN and partitioning WRZ

We put forward the rules of grading RN and partitioning WRZ (Tables 1,2) to determine the topological relationship and ensure that the codes for RN and WRZ are consistent. The L1 RN is rivers or inland rivers on the same continent that can flow into the same ocean. Accordingly, the L1 WRZ is defined as the zone composed of the basin where the L1 RN is located. The Sahara Desert, the largest desert in the world, covers more than 31.7% of the land area of Africa. It is precise because its area is too large, and the absence of RN that can flow to the ocean in this zone, as well as the extremely scarce precipitation in the region, that the surface may only have theoretical river networks but no natural rivers. Therefore, we defined a new type of L1 WRZ, the Sahara Desert zone. For L2 RN, we define them according to whether the drainage area of the exorheic rivers in the L1 RN is larger than 500000 km^2^ or the rivers that flow into the endorheic basin, which can be classified as the single river network (SRN, the drainage area is larger than 500000 km^2^) and the mixed river network (MRN, the drainage area is smaller than 500000 km^2^) and the endorheic river network (ERN). Accordingly, based on the L1 WRZ, the L2 WRZ is divided into four types, which are the single water resources zone (SWRZ), mixed water resources zone (MWRZ), endorheic water resources zone (EWRZ), the Sahara Desert water resources zone (SDWRZ). Among them, SWRZ refers to the basin of an exorheic river with a drainage area larger than 500000 km^2^ and the area on both sides of its estuary; MWRZ refers to the zone composed of several basins of exorheic rivers with a total drainage area of smaller than 500000 km^2^ between two adjacent SWRZ. The RN at L3 to L7 is generated according to the corresponding thresholds. Their corresponding basins are called the WRZ at L3 to L7, respectively.

**Table 1:** Rules of grading RN at L1 to L7.

RN level	Rules of grading RN at L1 to L7
L1	The rivers flow into the same ocean or the same endorheic basin (excluding Greenland and Antarctica).
L2	The drainage area of the exorheic rivers is larger than 500000 km^2^ or the rivers that flow into the endorheic basin.
L3	The rivers flow into the L2 RN with a confluence area larger than 50000 km^2^ or flow into the ocean alone.
L4	The rivers flow into the L3 RN with a confluence area larger than 10000 km^2^ or flow into the ocean alone.
L5	The rivers flow into the L4 RN with a confluence area larger than 1000 km^2^ or flow into the ocean alone.
L6	The rivers flow into the L5 RN with a confluence area larger than 100 km^2^ or flow into the ocean alone.
L7	The rivers flow into the L6 RN with a confluence area larger than 50 km^2^ or flow into the ocean alone.

**Table 2:** Rules of partitioning WRZ at L1 to L7.

WRZ level	Rules of partitioning WRZ at L1 to L7
L1	The zone where the L1 river is located (excluding Greenland and Antarctica) and the Sahara Desert zone.
L2	The zone where the L2 river is located (including the single water resources zone (SWRZ), the mixed water resources zone (MWRZ), the endorheic water resources zone (EWRZ), and the Sahara Desert water resources zone (SDWRZ)).
L3	The basin where the L3 river is located with an area larger than 50000 km^2^.
L4	The basin where the L4 river is located with an area larger than 10000 km^2^.
L5	The basin where the L5 river is located with an area larger than 1000 km^2^.
L6	The basin where the L6 river is located with an area larger than 100 km^2^.
L7	The basin where the L7 river is located with an area larger than 50 km^2^.

### Generate RN and WRZ

The first step in generating RN and WRZ is using the L1 WRZ in GRNWRZ V1.0 to determine the initial boundaries based on the continent where it is located and the ocean in which it flows. To ensure accuracy, we expand the initial boundaries by 50 km to form a buffer. Then, the initial boundaries are further subdivided and formed into L2 WRZ according to the rules (Tables 1,2) of partitioning WRZ. Finally, L2 WRZ is used as the basic unit to complete the simultaneous generation of grading RN and partitioning WRZ at L3-L7 by following the thresholds (Tables 1,2) and tracing its source backward from the estuary.

We use two DEM data sets (the SRTM and the ASTER GDEM) to generate RN and WRZ, with spatial resolutions of 90 m (for SRTM) and 30 m (for ASTER GDEM). The SRTM data (publicly available on ‘https://earthexplorer.usgs.gov/’) was measured by the National Aeronautics and Space Administration (NASA), the National Geospatial-Intelligence Agency (NGA), and the German and Italian space agencies. The ASTER GDEM data (publicly available on ‘https://search.earthdata.nasa.gov/’) was developed by NASA and Japan’s Ministry of Economy, Trade, and Industry (METI). The SRTM only covers the global area between 60°N and 56°S. To ensure the integrality of the global RN and WRZ, we resampled the ASTER GDEM to upscale its spatial resolution from 30 m to 90 m and then spliced it with the SRTM to form a complete global DEM.

#### Determine WRZ at level 2

We use the L1 WRZ in GRNWRZ V1.0 to determine the initial boundaries and expand it by 50 km to form a buffer. Then, we use them to generate basins and select the complete zone with an area larger than 500000 km^2^ as SWRZ. Next, we select the zone composed of several basins of exorheic rivers with a total drainage area of smaller than 500000 km^2^ between two adjacent SWRZ as MWRZ. For the endorheic basin, we obtain L2 WRZ through the methods proposed in our previous research^28^. Specifically, we expand the boundary of the endorheic L1 basins in GRNWRZ V1.0 by 50 km to form a buffer. Then, we remove the elevation data in the lake area by the land use data^29^, creating an outlet for flow for the internally-draining basin, and then we get sub-basins by ArcGIS hydrologic tools. Finally, we combine flow accumulation to dissolve all sub-basins to form L2 EWRZ and SDWRZ. As a fundamental computing unit, the L2 WRZ can perform high-efficiency calculations within limited computer memory, reducing the calculation pressure and the uncertainty of the calculation process caused by hardware limitations.

#### Generate preparation data for RN and WRZ

The RN in the region with high altitude and undulating terrain can easily be generated by ArcGIS, while in the region with low elevation and flat terrain or the endorheic basin, it is difficult for the RN to get the correct flow direction due to flat terrain or lack of outlets. Therefore, we combined the digital RN in GRNWRZ V1.0^24^ and the water bodies of the land use data^29^ and used the stream burning^24,30^ method to modify the original DEM data. The modified DEM data can ensure that the generated river conforms to the natural river morphology. Fills sinks are required to remove small imperfections in the modified DEM data. Then hydrological data, such as flow direction and flow accumulation, were generated by the filled DEM based on the hydrological analysis tools of ArcGIS.

#### Determine the identification number for RN and WRZ at levels 3 to 7

**Fig. 2:**
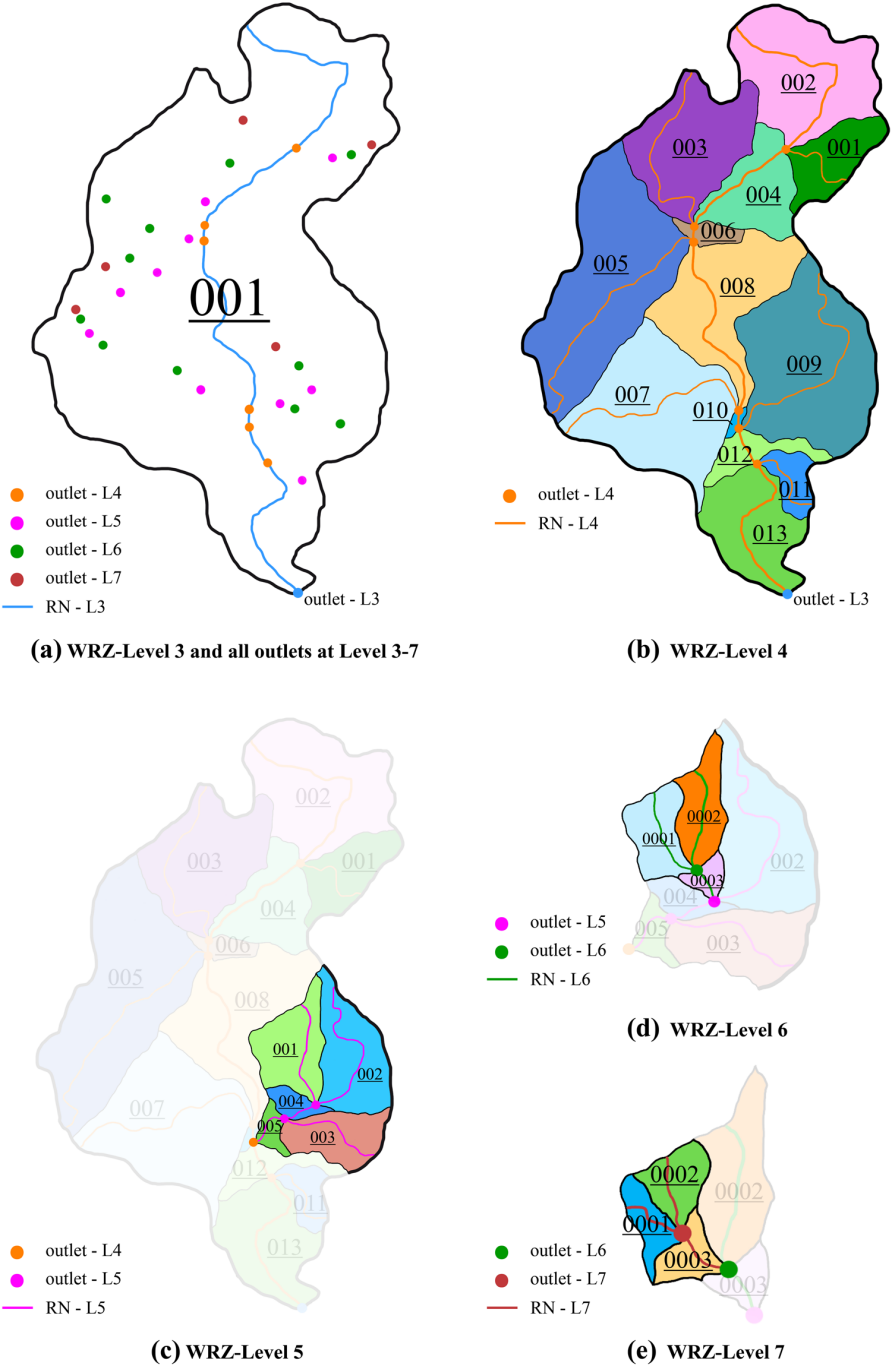
Determining the topological relationship and the identification number (IN) of river networks (RN) and water resources zones (WRZ) at L3-L7. (**a**) The L3 WRZ and all L3-L7 outlets in it. (**b**) L4 RN and its corresponding L4 WRZ are generated from all L4 outlets. (**c**) In the L4 WRZ coded as ‘009’, L5 RN and its corresponding L5 WRZ are generated from all L5 outlets. (**d**) In the L5 WRZ coded as ‘001’, L6 RN and its corresponding L6 WRZ are generated from all L6 outlets. (**e**) In the L6 WRZ code as ‘0001’, L7 RN and its corresponding L7 WRZ are generated from all L7 outlets.

We set the identification number (IN) to identify the RN and the WRZ from L3 to L7. The IN from L3 to L5 consists of three digits ranging from 0 to 999(Fig. 2a–c). The IN from L6 to L7 comprises four digits, and the value range is from 0 to 9999 (Fig. 2d,e). If the IN of WRZ is ‘000’ or ‘0000’, it means that the area of the WRZ does not conform to the threshold for the corresponding level.

To ensure that the RN and the WRZ from L3 to L7 have a correct topological relationship, we set IN of each tributary and corresponding WRZ to be an odd number and IN of each main stream reaches and corresponding WRZ to be an even number. The IN of the last main stream connected to the outlet of each level of WRZ increases successively (Fig. 2b).

**Fig. 3:**
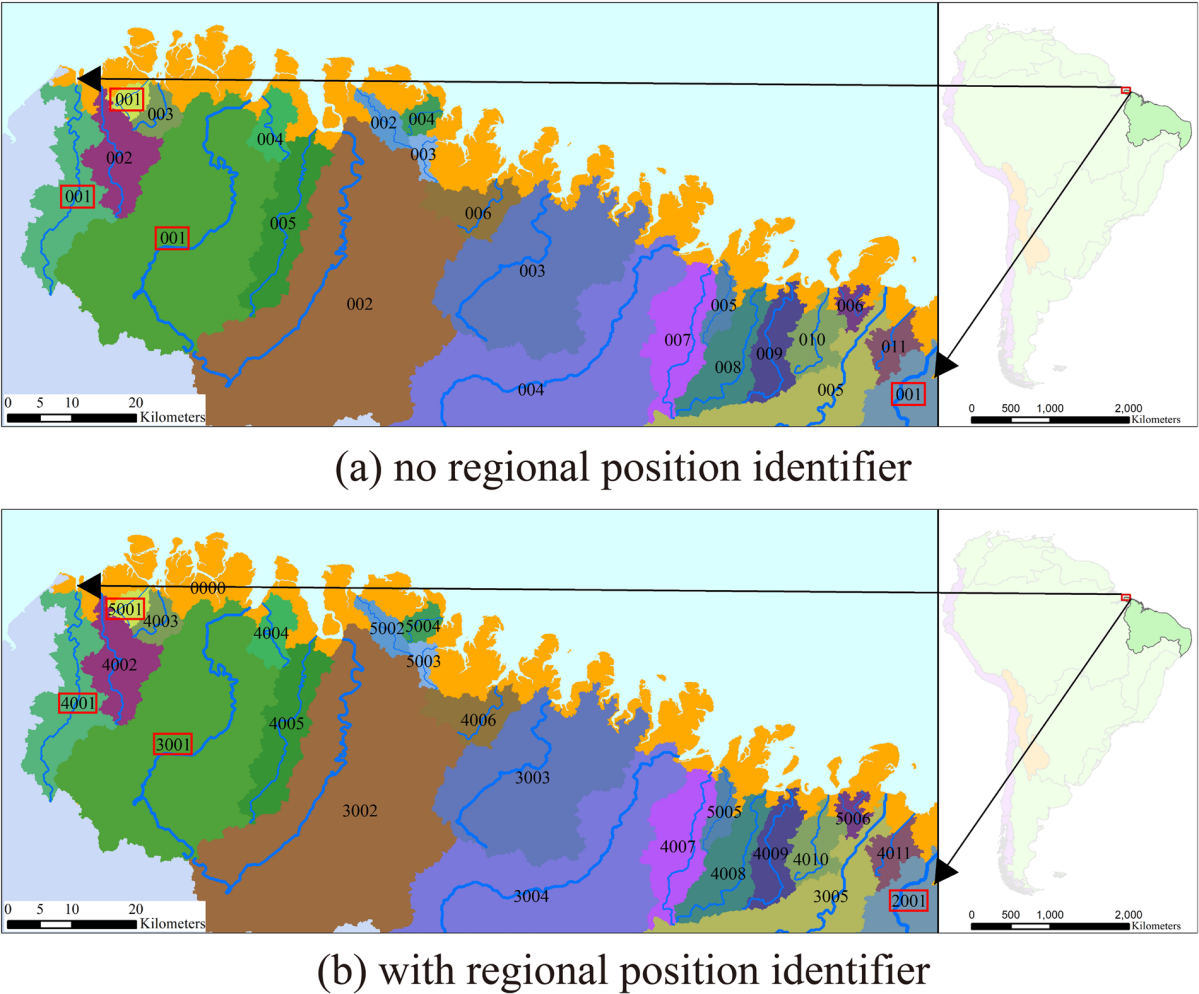
The regional position identifier (RPI) of the mixed water resources zone (MWRZ) around the east coast of Marajo Bay in South America. (**a**) With no RPI, many repeated identify numbers (IN) appear multiple times, making it challenging to encode WRZ and impossible to determine the level of RN and WRZ. For example, four ‘001’ in the red box. (**b**) With RPI, the hierarchy is clear. The ‘2001’/‘3001’/‘4001’/‘5001’ indicates that the zone is the first level 4/5/6/7 WRZ within this L2 WRZ, and its area is larger than 10000/1000/100/50 km^2^, respectively.

It was relatively simple for SWRZ, EWRZ, and SDWRZ to generate and code RN and WRZ because of the fixed corresponding outlets. However, there are often multiple WRZ with the same threshold for the coastal region, which will cause confusion in the IN and destroy the integrity of IN in the whole region (Fig. 3a). Therefore, we set up a regional position identifier (RPI) to further indicate the level and location of each RN and WRZ (Fig. 3b). The RPI is composed of four digits in the format of XZZZ. X represents each threshold, with a value range from 1 to 5, representing the level for grading RN and partitioning WRZ from L3 to L7. ZZZ is a sequential number ranging from 1 to 999, which indicates the number of RN and WRZ under the current threshold. The ‘0000’ shows a continuous region composed of sub-basin whose areas are all smaller than 50 km^2^.

#### Generate the RN and WRZ at levels 3 to 7

**Fig. 4:**
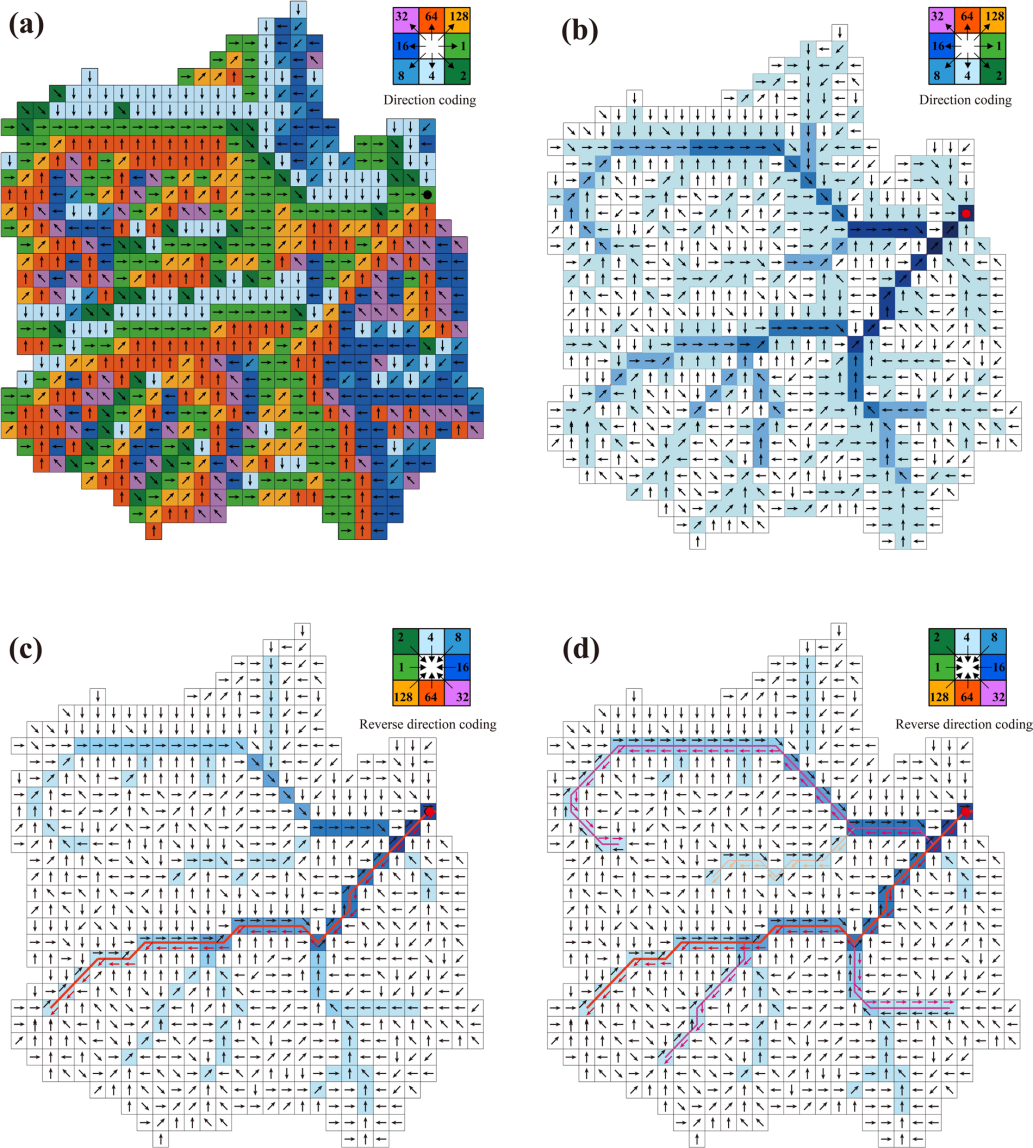
Reversely determine the source of main stream reaches, main stream reaches and tributaries in a watershed. (**a**) shows flow direction. (**b**) shows flow direction and flow accumulation greater than 0. (**c**) The red arrow indicates searching the trunk stream in the reverse direction. (**d**) The pink and orange arrows represent the reverse search for tributaries.

We determined the level of outlets by selecting the flow direction and the flow accumulation of each L2 WRZ and extracting the flow accumulation corresponding to each outlet according to the spatial sequence from west to east and north to south, and then the relationship between outlets and thresholds are judged one by one (Fig. 2a). Next, we take the outlets of each level as the starting point, refer to the deterministic eight neighbors (D8) flow model^31,32^, and trace back pixel by pixel through flow direction data from the outlet to the river source(Fig. 4a–c). When multiple rivers merge into the same point simultaneously, the river segment with larger flow accumulation is taken as the main stream reaches to search upstream again, ensuring that we can find sources of each river (Fig. 4d). Finally, RN and WRZ from L3 to L7 are generated according to the outlets of each level and the flow direction data (Fig. 2a–f). It should be noted that there are two types of mismatches between the watershed size and the threshold that cannot be evitable. In one case, if many tributaries flow into a small section of the main stream reaches, several small watersheds would be on the corresponding WRZ of the main stream reaches. In the other case, if no tributaries flow into the section of the main stream reaches with a long-distance, there would be a larger watershed on the corresponding WRZ of the main stream reaches. We strictly follow the hierarchical partition threshold to generate RN and WRZ from L3 to L7 to minimize the occurrence of both situations as much as possible.

### Determine the codes of RN and WRZ

#### Determine coding rules

**Fig. 5:**
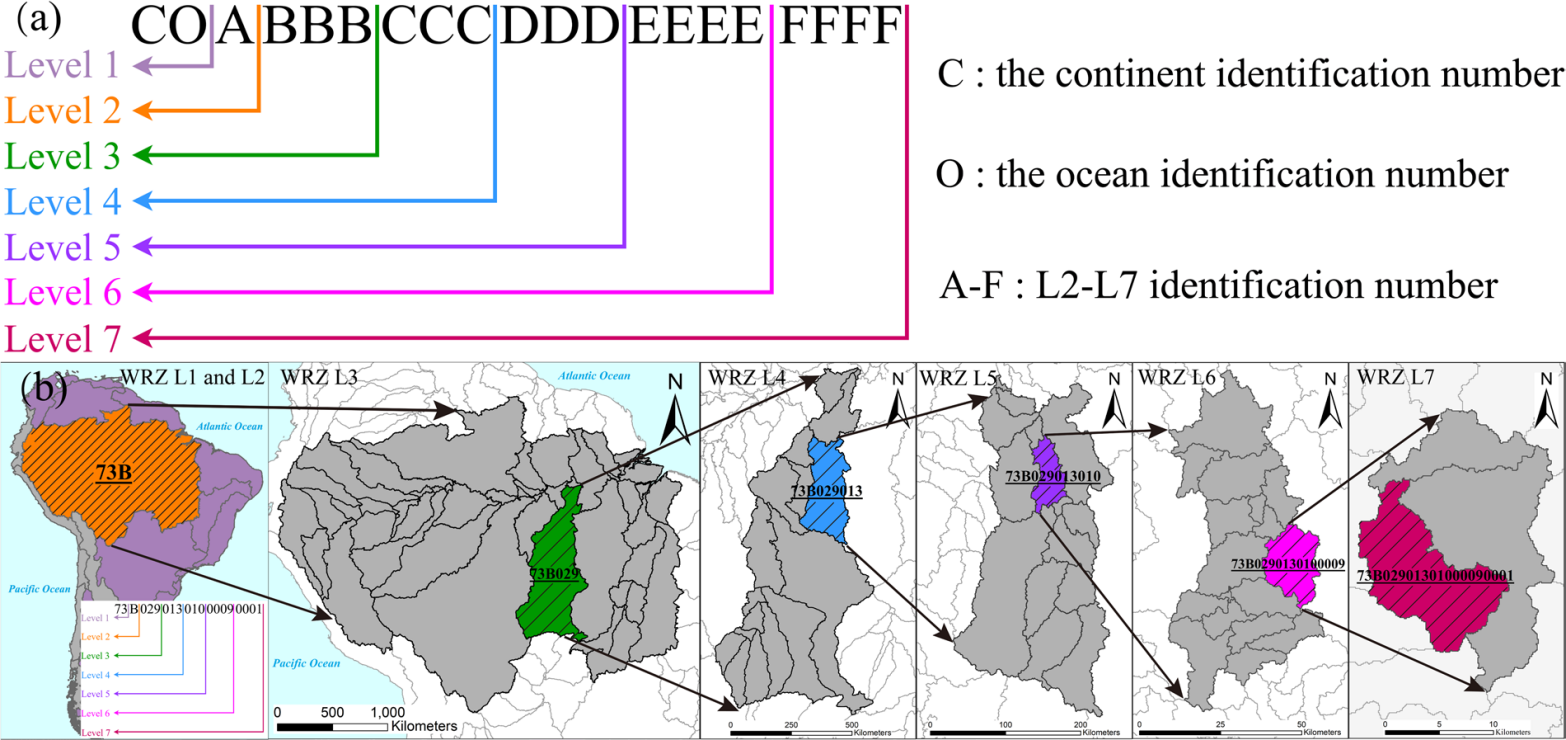
Interpretation diagram of codes. (**a**). Interpretation of the code at each level. The character ‘C’ indicates the continent identification number (IN). The character ‘O’ indicates the ocean IN. The characters ‘A’-‘F’ indicates the IN at L2-L7, respectively. The number of times they recur indicates the length of the IN. (**b**) The Amazon basin in South America is an example to show the correspondence between codes and WRZ at each level.

**Fig. 6:**
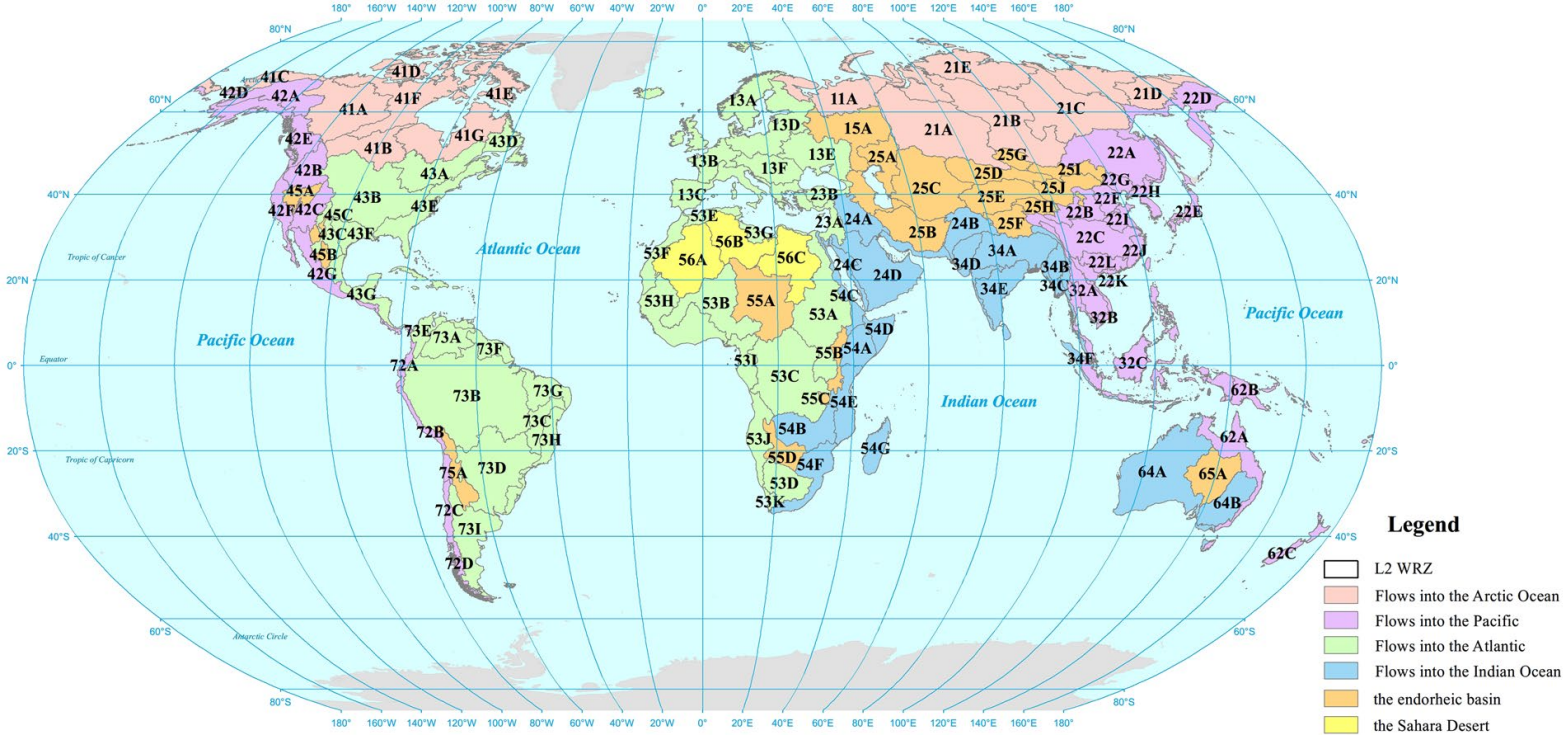
The global WRZ at level 2. The first character is the continent identification number (IN) (Europe = 1, Asia I = 2, Asia II= 3, North America = 4, Africa = 5, Oceania = 6, South America = 7. Considering the basin’s integrity and the area deformation, we divided Asia into Asia I and Asia II by referring to the Tropic of Cancer). The second character is the river outflow IN (Arctic Ocean = 1, Pacific = 2, Atlantic = 3, Indian Ocean = 4, endorheic basin = 5, Sahara Desert = 6). The third character is the IN of L2 WRZ.

The RN and WRZ codes for L1 to L7 consist of 2 to 20 characters (Fig. 5a). The L1 code consists of two characters. The first character is the continent IN, indicating which continent the RN and WRZ are located in (Europe = 1, Asia I = 2, Asia II = 3, North America = 4, Africa = 5, Oceania = 6, South America = 7. Considering the basin’s integrity and the area deformation, we divided Asia into Asia I and Asia II by referring to the Tropic of Cancer). The second character of the L1 code is the river outflow IN, indicating which ocean or zone the RN in the WRZ flow into (Arctic Ocean = 1, Pacific = 2, Atlantic = 3, Indian Ocean = 4, endorheic basin = 5, Sahara Desert = 6). The L2 code consists of three characters. The first two characters are the L1 code. The third character is the IN of L2 WRZ. The SWRZ, the CWRZ, the EWRZ, and the SDWRZ are successively represented by capital English letters (A, B, C,…) in a spatial order from west to east and north to south. The spatial distribution of all L2 WRZ is shown in Fig. 6. Starting from L3, the code of each level consists of the previous level code and the IN of the current level. L3/L4/L5 consists of 6/9/12 characters, the first 3/6/9 characters are coded at L2/L3/L4, and the 4–6/7–9/10–12 characters are the IN of the L3/L4/L5. L6/L7 consists of 16/20 characters, the first 12/16 characters are the L5/L6 code, and the 13–16/17–20 characters are the IN of L6/L7. Using this coding rule, anyone can easily obtain the topological relation of various RN and the WRZ through simple character interception.

As shown in Fig. 5b, the code represents RN and WRZ at L1 to L7 in South America. Among the code, ‘73’ is a code of L1 RN and WRZ, indicating the zone where the rivers flowing into the Atlantic Ocean are located in South America. ‘73B’ is one code of L2 RN and WRZ, representing the second independent zone from west to east and north to south in ‘73’, namely the Amazon basin. ‘73B029’ is one code of L3 RN and WRZ, it is a watershed where the 15th tributary of the ‘73B’ WRZ is located, and its drainage area is larger than 50000 km^2^. ‘73B029013’ is one code of L4 RN and WRZ, it is a watershed where the seventh tributary of the ‘73B029’ WRZ and its drainage area is larger than 10000 km^2^. ‘73B029013010’ is one code of L5 RN and WRZ, it is a watershed where the fifth trunk stream reaches of the ‘73B029013’ WRZ is located with a drainage area larger than 1000 km^2^. ‘73B0290130100009’ is one code of L6 RN and WRZ, it is a watershed where the fifth tributary of the ‘73B029013010’ WRZ is located, and its drainage area is larger than 100 km^2^. ‘73B02901301000090001’ is one code of L7 RN and WRZ, it is a watershed where the first trunk stream reaches of the ‘73B0290130100009’ WRZ is located with a drainage area of more than 50 km^2^.

#### Generate codes

Considering the IN at all levels, we form a complete code based on following the rules of grading RN and partitioning WRZ and not destroying the river system connections. However, each basin may contain several WRZ of multiple levels in the CWRZ. This situation can make one code refer to multiple RN and WRZ simultaneously, which will cause confusion in river system connections and undermine the integrity of the WRZ. Therefore, we sort the IN in ascending order and combine with RPI to complete the coding of the RN and WRZ at all levels to maintain the integrity of the topological relationship.

## Data Records

Through the above methods, we generated global WRZ from L1 to L7. The number of WRZ at each level was 24, 120, 965, 5574, 56769, 567238 and 1415316, respectively(Table 3).

**Table 3:** Statistical table of the number of WRZ from L1 to L7 in the world.

Level	Europe	Asia*	North America	Africa	Oceania	South America	Global
L1	3	7	4	4	3	3	24
L2	8	43	24	25	6	14	120
L3	71	289	166	233	73	133	965
L4	403	1856	863	1287	347	818	5574
L5	4099	19291	9260	12944	3756	7419	56769
L6	42253	193491	93524	126167	37201	74602	567238
L7	103773	484985	231081	314993	93264	187222	1415316

* Asia I and Asia II are merged into Asia.

This data set is available within Figshare^33^ and consists of two parts. Part I is seven zip files, which are RN vector data of the world’s continents (except Greenland and Antarctica) with the names ‘Global_River_Networks_and_corresponding_Water_Resources_Zones_divisions 2.0 (GRNWRZ2) _River_Networks (RN)_XX_Shapefile’ (X is the name of each continent). These RN files with a shapefile file format (.shp), and their attribute table mainly includes attribute information such as ‘FID’, ‘level’, and ‘length’(Table 4). Part II is also seven zip files with the names ‘Global_River_Networks_and_corresponding_Water_Resources_Zones_divisions2.0(GRNWRZ2)_Water_Resources_Zones(WRZ)_X_Shapefile’ (X is the name of each continent). They are the vector data of WRZ from L1 to L7 in all world continents (except Greenland and Antarctica). The format of these files is the same as RN vector data in Part I, and their attribute table contains four data labels: ‘FID’, ‘code’, ‘Area’, and ‘RPI’ (Table 5). The coordinates of all vector files are the WGS 1984 geographic coordinates.

**Table 4:** The attribute table of RN from L1 to L7.

Data label	Description
FID	The ID code of the river
level	The level of the river
Length	The length of the river (km)

**Table 5:** The attribute table of WRZ from L1 to L7.

Data label	Description
FID	The ID of WRZ
code	The code of WRZ
Area	Area (km^2^)
RPI	The regional position identifier of WRZ (only levels from 3 to 7)

## Technical Validation

### Improvements of GRNWRZ V2.0 compared to GRNWRZ V1.0

We first demonstrate the new hydrography dataset’s improvement by comparing it against GRNWRZ V1.0.

**Fig. 7:**
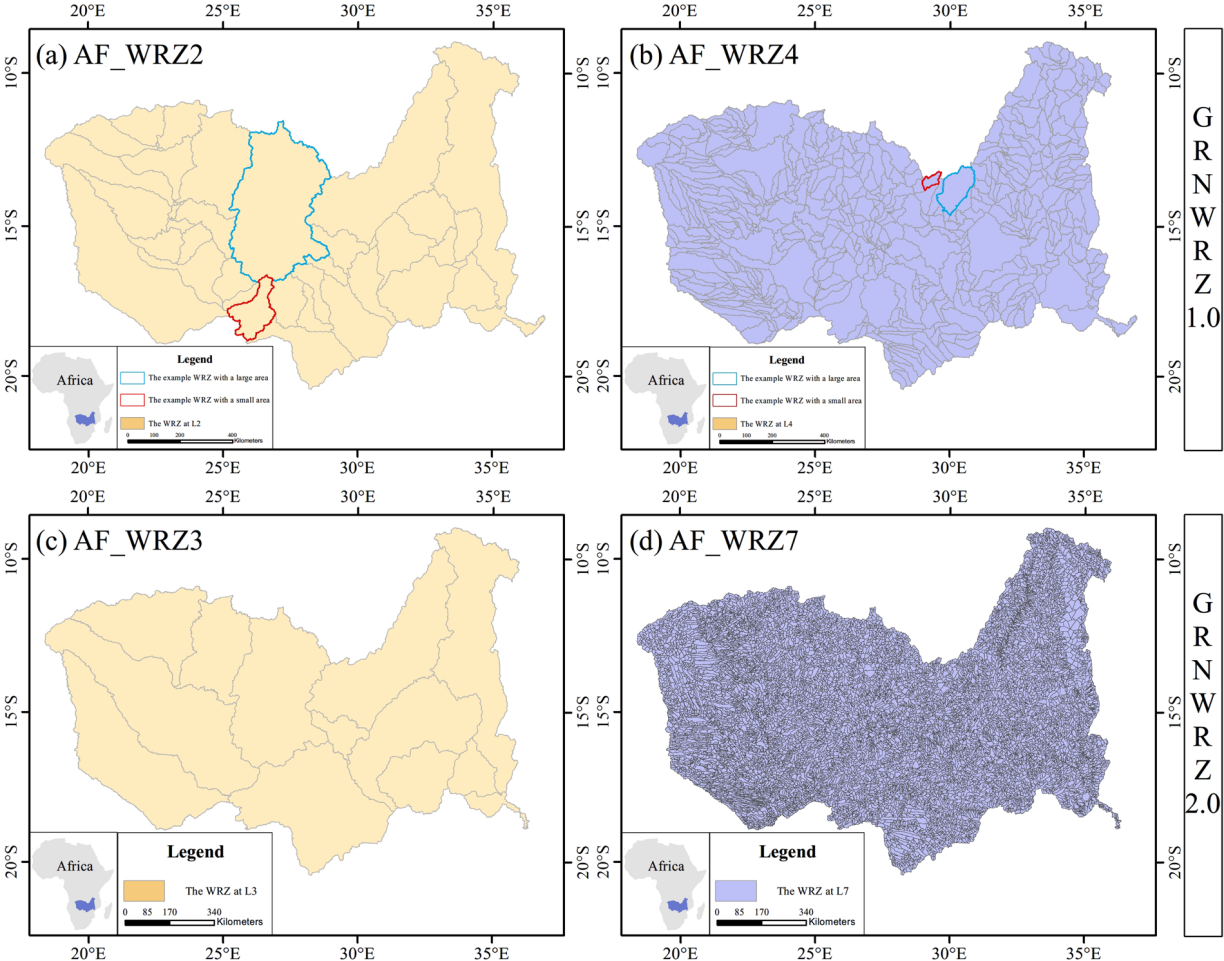
The difference in the areas of the WRZ in GRNWRZ V1.0 and GRNWRZ V2.0, with the Zambezi River basin as an example. In GRNWRZ V1.0, the coefficient of variation in the area of (**a**) the L2 WRZ basins and (**b**) L4 WRZ basins are larger ((**a**) 1.231, (**b**) 1.469) compared to (**c,d**) GRNWRZ V2.0 ((**c**) 0.575, (**d**) 0.894).

In GRNWRZ V1.0, they divided L2 WRZ (V1L2WRZ), L3 WRZ (V1L3WRZ) and L4 WRZ (V1L4WRZ) with three thresholds of 10000 km^2^, 1000 km^2^ and 100 km^2^, respectively. In GRNWRZ V2.0, we used five thresholds of 50000 km^2^, 10000 km^2^, 1000 km^2^, 100 km^2^ and 50 km^2^ to divide L3 WRZ (V2L3WRZ), L4 WRZ (V2L4WRZ), L5 WRZ (V2L5WRZ), L6 WRZ (V2L6WRZ), and L7 WRZ (V2L7WRZ), respectively. Since the two datasets have different levels, we look for similar or corresponding thresholds in the respective partitioning rules to specify the levels for comparison. That is, V1L2WRZ corresponds to V2L3WRZ and V2L4WRZ, V1L3WRZ corresponds to V2L5WRZ and V1L4WRZ corresponds to V2L6WRZ and V2L7WRZ. Overall, compared with V1.0, the mean and median of the area of each level’s WRZ in GRNWRZ V2.0 are closer to the thresholds (Table 6). In addition, the standard deviation and the coefficient of variation representing their degree of dispersion are smaller. These indicate that there is no particularly significant difference in the areas of the WRZ in GRNWRZ V2.0 (Fig. 7).

**Table 6:** The comparison of WRZ division rationality.

Dataset	Level	Threshold (km^2^)	Number of WRZ	Median (km^2^)	Mean (km^2^)	Standard deviation (km^2^)	Coefficient of variation
GRNWRZ V1.0	L2	10000	1672	26752.71	80471.24	154504.60	1.920
	L3	1000	22871	1827.88	5882.94	25246.28	4.291
	L4	100	60837	1393.72	2211.77	12542.70	5.671
GRNWRZ V2.0	L3	50000	965	80081.56	136929.20	191956.80	1.402
	L4	10000	5574	15555.60	23705.90	49306.95	2.080
	L5	1000	56769	1589.73	2327.62	6381.095	2.741
	L6	100	567238	161.75	232.95	541.70	2.325
	L7	50	1415316	74.97	93.36	83.41	0.893

**Fig. 8:**
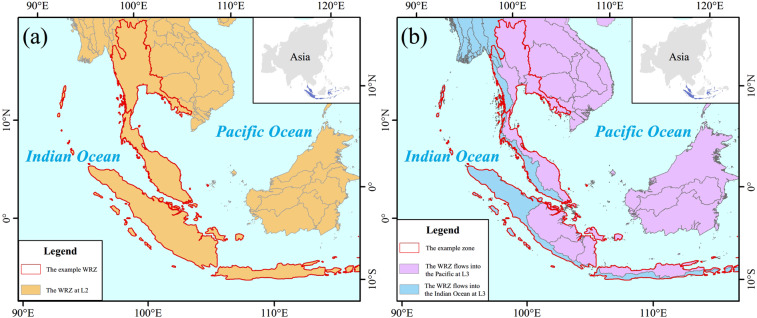
The WRZ is divided according to flows into the same ocean. (**a**) The L2 WRZ is not divided according to the final flow direction in GRNWRZ V1.0. (**b**) The L3 WRZ divided flows into the Pacific (blue part**)** and the Indian Ocean (purple part) according to the final flow direction in GRNWRZ V2.0.

When conducting water resources statistics on a global scale, it is very necessary to identify the ocean to which the exorheic rivers ultimately flow. GRNWRZ V1.0 does not consider this issue(Fig. 8a). However, we added the division by ocean boundary condition in GRNWRZ V2.0. It ensures that the water resources of exorheic rivers on the land surface have correct ascription (Fig. 8b) and the applicability of GRNWRZ V2.0.

### Compared with HydroSHED and HDMA

We chose HydroSHED^9^ and HDMA^11^ databases for accuracy comparison. They are two widely used global hydrological data sets in which the secondary products (HydroRIVERS^34^ and Stream layers) can provide us with an objective comparison. We refer to the comparison method in GRNWRZ V1.0 to compare the accuracy of our RN with HydroRIVERS and Stream layers. It should be noted that few rivers in Stream layers can meet the criteria for rivers at level 7 in GRNWRZ V2.0, so the rivers in Stream layers cannot completely cover the comparison points. Therefore, we only compare with HydroRIVERS at level 7. The comparison is made in the following way: Step 1. We randomly selected 400 rivers from L2-L7 RN of each continent, and three randomly points were generated at each river by ArcGIS (because the Level 1 RN consists of all Level 2 RN that flow into the same ocean, they are essentially the same rivers but divided by different criteria). Step 2. We imported these random points into Google Earth and manually marked the center point of natural rivers nearest to the random point. Step 3. We calculate the deviation distances from the center point obtained in step 2 to rivers in the other two data sets by ArcGIS. Then, we performed statistics in Excel to derive the comparison results.

In addition, since GRNWRZ V1.0 RN was obtained according to the natural rivers in Google Earth carefully and manually, we also evaluated the accuracy of RN by comparing with it. Since the RN’s level of GRNWRZ V1.0 is level 1 - level 4, which corresponds to our L2, L3-L5, and L6 RN, we only compared these four levels. We calculated the relative error between the river network in V2.0 and the river network in V1.0. The specific formula is as follows:

$$\delta =\frac{\left|{V}_{2}-{V}_{1}\right|}{{V}_{1}}\times 100 \% $$

Where *δ* is the relative error, *V*_2_ is the distance between the center point of natural rivers and the river network in GRNWRZ V2.0, and *V*_1_ is the distance between the center point of natural rivers and the river network in GRNWRZ V1.0. The distance between the center point of natural rivers and the river network is obtained in the same way as steps 1–3 mentioned above.

**Fig. 9:**
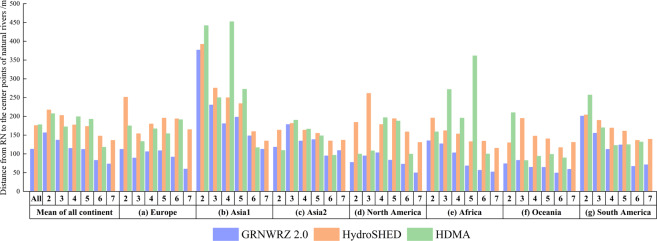
The mean deviation distance from RN to the center points of natural rivers in GRNWRZ V2.0, HydroSHED, and HDMA.

Overall, it can be seen from Fig. 9 that the RN of GRNWRZ V2.0 has shorter deviation distances. The deviation distance from the natural river center point in Google Earth to the RN of GRNWRZ V2.0 is 112.6 m, while the deviation distance to the HydroRIVERS is 175.52 m, and the deviation distance to the Stream layers in the HDMA is 177.64 m (level 2–6). Likewise, each level deviation distance is significantly shorter than in the other two data sets in the levels from 2 to 7. For each continent, the deviation distance of the RN of GRNWRZ V2.0 is significantly shorter than that of HydroRIVERS and Stream layers in the HDMA. Compared with HydroRIVERS, the mean deviation distance of the RN of GRNWRZ V2.0 decreased by 3.05 m to 166.38 m. Compared with Stream layers in the HDMA, the mean deviation distance of the RN of GRNWRZ V2.0 decreased by 0.59 m to 293.05 m, except for L6 RN in Asia I and L2 RN in Asia II, and L3 RN in Oceania. It should be pointed out that the spatial resolution of the DEM data we used is 90 m, which cannot wholly coincide with the centerline of the river with a width less than 90 m. Therefore, the DEM data with the higher spatial resolution is essential to generate RN in future work.

**Fig. 10:**
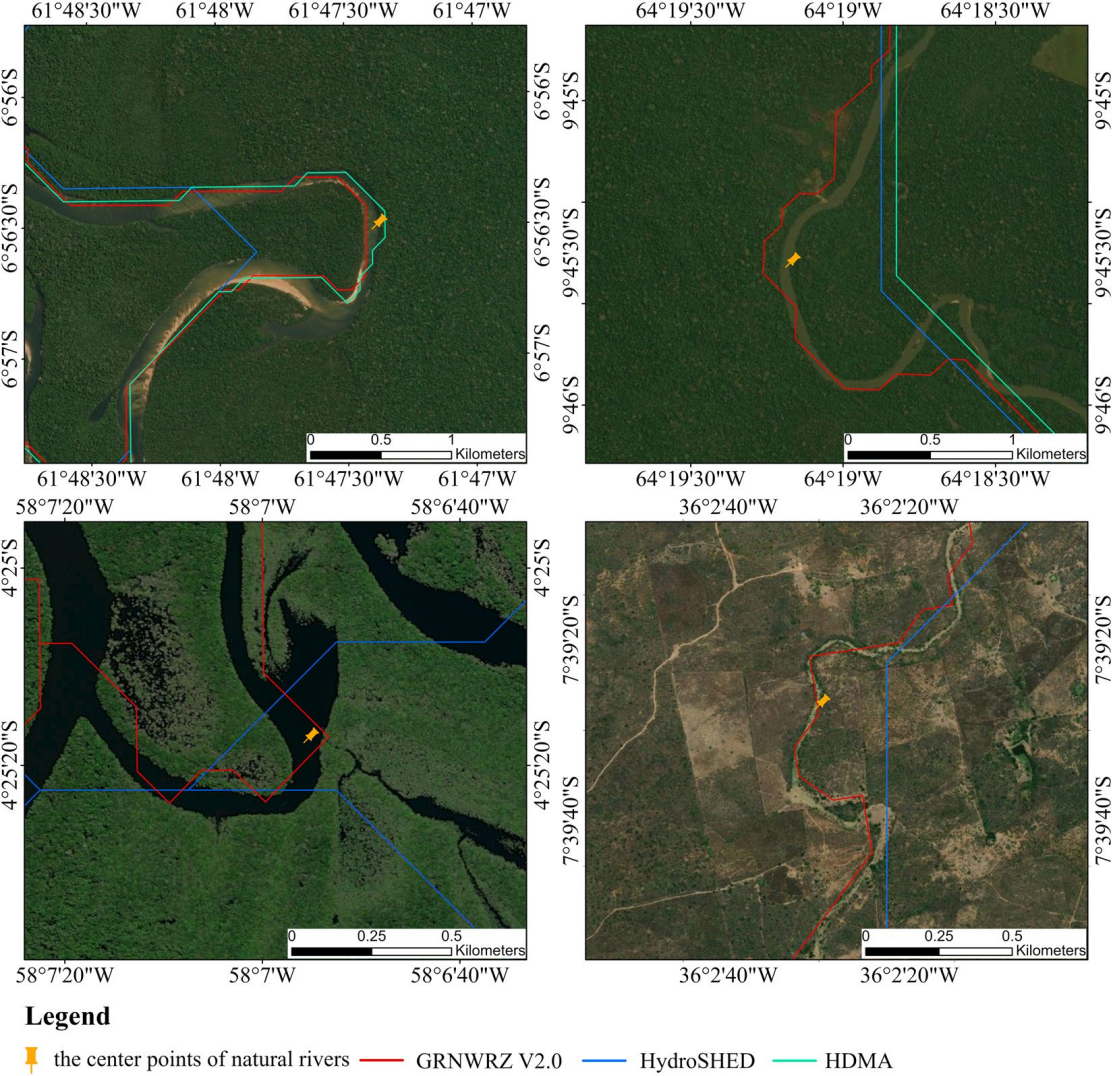
Comparison the RN among GRNWRZ V2.0, HydroSHEDS, and HDMA. (**a**) The river is the Dos Marmelos River, which is a tributary of the Madeira River in South America. (**b**) The river is the Jaci Paraná River, which is also a tributary of the Madeira River in South America. (**c**) The river is the Abacaxis River in South America. (**d**)The river is connected to the Paraíba River in South America.

Figure 10 shows that GRNWRZ V2.0 is more reasonable in river details, especially at the river’s bend. It can better fit the natural river and conform to the shape of natural river courses.

**Fig. 11:**
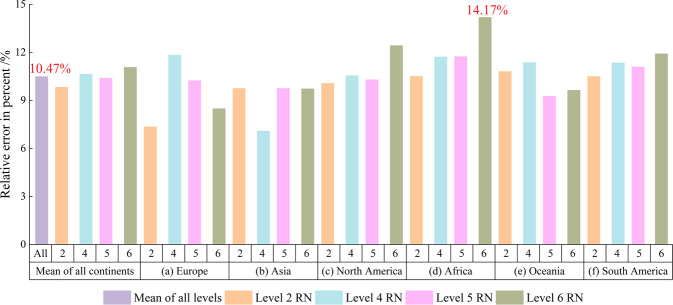
The relative error in percent of GRNWRZ V2.0 for GRNWRZ V1.0.

The comparison results in Fig. 11 show that the mean relative error of GRNWRZ V2.0 for GRNWRZ V1.0 is 10.47%, and the largest relative error is 14.17% which is in the L5 RN in Africa.

In summary, GRNWRZ V2.0 has several advantages: (1) the generated digital rivers are relatively accurate position compared with other datasets; (2) the WRZ and the corresponding RN were obtained using a more reasonable division method and more detailed division thresholds, which solves some problems in GRNWRZ V1.0 and can be better applied in hydrological simulations; (3) The new coding method can represent the topological relationships, which overcome coding upper limit in GRNWRZ V1.0 and can support more levels.

## Data Availability

The codes to construct the GRNWRZ V2.0 data set are available in Figshare^33^. There are two files and a folder: *01_Data Prepare.tbx* – Prepare hydrological data, including flow direction and flow accumulation. *02_RNWRZ* – Contain Fortran files, which are used to generate the RN and WRZ at levels from 3 to 7. *03_extract_RNWRZ.py* – Generate codes for the RN and WRZ. The ArcGIS Model(.tbx) and Python(.py) scripts are run in ArcGIS Pro (v2.4 or above vision). The remaining folder contains all Fortran files. It was compiled by Intel Parallel Studio XE 2018, installed in Visual Studio 2013. All software needs to be installed in the Windows 10 OS.
